# Torque teno virus dynamics during the first year of life

**DOI:** 10.1186/s12985-018-1007-6

**Published:** 2018-05-30

**Authors:** Elena A. Tyschik, Anastasiya S. Rasskazova, Anna V. Degtyareva, Denis V. Rebrikov, Gennady T. Sukhikh

**Affiliations:** 1 Kulakov National Medical Research Center for Obstetrics, Gynecology and Perinatology, Oparina 4, Moscow, 117513 Russia; 2 Pirogov Russian National Research Medical University, Ostrovityanova 1, Moscow, 117997 Russia

**Keywords:** Torque teno virus, Transfusion-transmitted virus, TTV, Viral load dynamics, Neonatal period, Infants, TORCH infections

## Abstract

**Background:**

Torque teno virus is a small chronically persisting circular negative ssDNA virus reaching near 100% prevalence. It is reported to be a marker for immune function in immunocompromised patients. The possibility of vertical maternal-fetal transmission remains controversial but incidence rate of TTV DNA in children increased with age. TTV dynamics well studied for allogeneic hematopoietic stem cell transplantation as a predictor of post-transplant complications but there is no viral proliferation kinetics data for other patient groups or healthy individuals. The aim of this study was to determine TTV dynamics during the first year of life of healthy infants.

**Methods:**

Ninety eight clinically healthy breastfeeding infants (1–12 months of age) were analyzed by quantitative PCR for the whole blood TTV load with the test sensitivity of about 1000 viral copies per milliliter of blood (total number of samples including repeatedly tested infants was 109).

**Results:**

67% of all analyzed samples were TTV-positive demonstrating significant positive correlation between age and TTV load (*r* = 0.81, *p* < 0.01).

**Conclusions:**

This is the first study to suggest that viral load increases during the first year of life reaching a plateau after 6 months with strong proliferation for the first 60 days. Our data well correlates with TTV dynamics in patients following allogeneic hematopoietic stem cell transplantation.

## Background

Torque teno virus (TTV) is a small chronically persisting circular negative ssDNA virus reaching near 100% prevalence [1, 2]. TTV is transmitted in all ways including contact and respiratory [3]. It was suggested that presence of TTV can cause several diseases such as acute respiratory diseases [4], liver diseases [5, 6] and cancer [7], but this data did not have any convincing support. It is reported to be a marker for immune function in immunocompromised patients [8].

The routes of mother-to-child transmission of TTV have not been fully elucidated and the possibility of vertical maternal-fetal transplacental transmission remains controversial [9–20]. Also, several authors demonstrated that incidence rate of TTV DNA in children increased with age [13, 15, 19], but there is no information about the viral load during the first months of life.

TTV dynamics well studied for allogeneic hematopoietic stem cell transplantation as a predictor of post-transplant complications [21, 22]. But there is no viral proliferation kinetics data for other patient groups or healthy individuals.

The aim of this study was to determine TTV dynamics during the first year of life of healthy breastfeeding infants.

## Methods

### Patients and blood samples collection

This prospective single-center study included 98 clinically healthy breastfeeding infants (1–12 months of age, number per month as 9; 6; 13; 8; 11; 14; 6; 9; 10; 6; 4; 2 accordingly). 10 infants were tested repeatedly (2 or 3 times), so the total number of samples was 109. The exclusion criteria were as follows: any infectious or genetic disease, any immunological deviations, voluntary refusal of research. Two separate aliquots of each capillary blood sample were collected into Microvette 200 K3EDTA (Sarstedt, Germany) between June 2017 and January 2018 at the Kulakov National Medical Research Center for Obstetrics, Gynecology, and Perinatology (Moscow, Russia). Samples were stored at − 20 °C for 1–7 days until DNA extraction.

### DNA extraction

DNA was extracted from 50 μl aliquots of thawed whole blood using a standard commercial silica-sorbent kit for DNA extraction from body fluids (Probe-GS DNA Extraction Kit, DNA-Technology, Russia). To prevent exogenous contamination, DNA isolation was performed in a separate DNA extraction room (Zone 1). To prevent cross-contamination of the samples, all procedures were carried out in the UV-equipped PCR-box using sterile disposable tubes and aerosol-resistant tips.

### TTV quantification

qPCR was performed using the DTprime Real-Time PCR Cycler (DNA-Technology, Russia) as described in [1], with the test sensitivity of about 1000 viral copies per milliliter of blood. qPCR of the unique human genome fragment (in a separate PCR tube) was used as DNA extraction control. To prevent PCR contamination by previous reactions or biological samples, the reactions were combined using aerosol-resistant tips in UV-equipped PCR-box in a separate PCR-preparation room (Zone 2). Also, no electrophoresis of TTV PCR products or other procedures that would require PCR-tube opening were performed in the building. All the negative controls and surface washings were negative.

### Data analysis

qPCR data were analyzed using the DTprime Real-Time PCR Cycler Software v.7.7 (DNA-Technology, Russia). Microsoft Office Excel 2016 (Microsoft Corporation, USA) and GraphPad Prism 6 (GraphPad Software, USA) were used for statistical analysis.

## Results

**Fig. 1:**
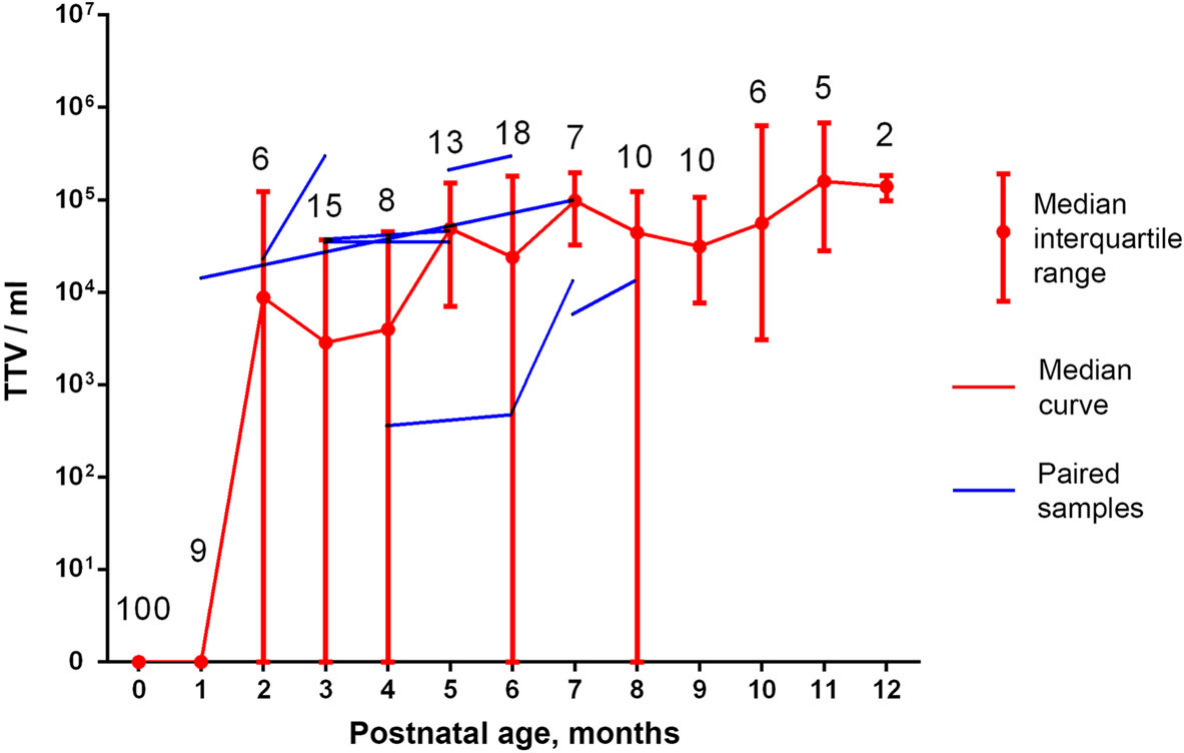
Torque teno virus dynamics during the first year of life. Data for 0 months from [20]. Numbers correspond to sample size

TTV whole blood viral load was quantified for 98 infants at 1–12 months of age (see. Fig. 1). Because of logistic difficulties only 10 infants were tested repeatedly (2 or 3 times) (blue lines at Fig. 1) and only several mother-child pairs were examined for TTV load (data not shown). No breast milk samples were tested.

67% of all analyzed samples were TTV-positive (higher than 10^3^ copies per 1 mL of whole blood as a test sensitivity) with median 5 × 10^4^ viral genomes per 1 mL (range of median values: 0–1,6 × 10^5^) demonstrating significant positive correlation between age and TTV load (*r* = 0.81, *p* < 0.01).

For 10 repeatedly tested infants 3 did not show TTV for both tests, 1 was unchanged and other 6 show more TTV in a second analysis (see Fig. 1).

## Discussions

Despite more than twenty years of TTV research, the routes of mother-to-child transmission have not been fully elucidated and the possibility of transplacental TTV transmission remains controversial [9–20]. Some authors demonstrate the absence of TTV in cord blood or baby blood after delivery [10, 13, 20], but other show 13.8–48.1% of TTV-DNA positive cord blood samples [9, 11, 14, 18]. Such differences can be explained by the low sensitivity of PCR (for studies where the virus did not detected) or by PCR-product contamination (since the cord blood TTV was usually detected by contamination-friendly Nested-PCR technique). In any case, it can be argued that even if the virus passes the transplacental barrier, the cord blood viral concentration is very low and does not depend on the mothers TTV load [20].

The major site of TTV replication is lymphocytes [23–25] and the whole blood TTV load approximately 100 times higher than plasma samples [20]. Consequently, we decided to measure TTV load in the whole blood (instead of plasma or serum) to get more sensitive approach.

**Fig. 2:**
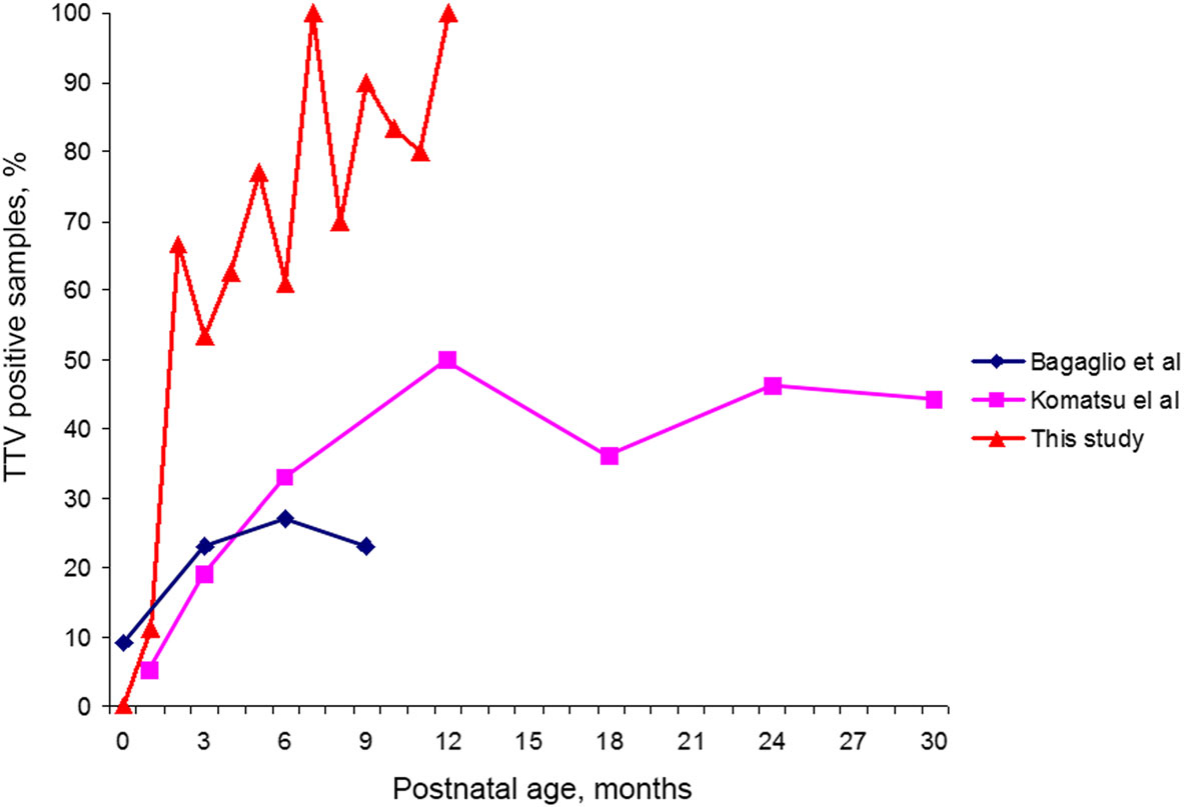
Percent of TTV DNA positive infants during the first months of life. Data from [15, 19] based on serum analysis

Bagaglio et al. and Komatsu et al. demonstrate increasing of the percent of TTV DNA positive infants during the first months of life by serum analysis [15, 19]. Our whole blood results correlates with previous serum data, expectantly showing a greater percentage of positive samples (see Fig. 2).

Breast milk is often positive for TTV (23.3–67.3%) [13, 14, 26] and it has been suggested to be one of the major routes of Torque teno virus transmission for babies. So, the newborn TTV progression may be a consequence of the mother’s breast milk TTV or immune system changes during the neonatal period.

## Conclusions

This is the first study to suggest that TTV viral load increases during the first months of healthy infants development reaching a plateau after 3–6 months with strong proliferation for the first 60 days. Fast viral load increasing correlates with previous data on TTV DNA prevalence. Also, neonatal TTV dynamics is similar to TTV proliferation in patients following allogeneic hematopoietic stem cell transplantation, demonstrating the possible similarity of intracellular mechanisms of viral progression.

## Abbreviations

PCR: Polymerase chain reaction
qPCR: Quantitative polymerase chain reaction
ssDNA: Single stranded deoxyribonucleic acid
TTV: Torque teno virus
UV: Ultraviolet
